# PNI, CONUT, and GNRI for predicting treatment failure in peritoneal dialysis-associated peritonitis: a two-center retrospective study

**DOI:** 10.3389/fnut.2026.1923256

**Published:** 2026-08-14

**Authors:** Yafeng Zhang, Guangxu Mao, Jing Yao, Bin Wu, Yue Zhou

**Affiliations:** 1 Department of Public Health, Affiliated Hospital of Jiangsu University, Zhenjiang, Jiangsu, China; 2 Department of Infection Management, Xinghua People's Hospital Affiliated to Yangzhou University, Xinghua, Jiangsu, China; 3 Department of Nephrology, Nanjing First Hospital, Nanjing Medical University, Nanjing, Jiangsu, China

**Keywords:** controlling nutritional status, geriatric nutritional risk index, peritoneal dialysis-associated peritonitis, prognostic nutritional index, treatment failure

## Abstract

**Background:**

Peritoneal dialysis-associated peritonitis (PDAP) is one of the common causes of technique failure in patients undergoing peritoneal dialysis. It may also affect long-term survival. Early identification of patients with a high risk of treatment failure is therefore important in clinical practice. Malnutrition has been reported to be related to poor outcomes in PDAP, but the predictive value of different nutritional scores has not been fully compared.

**Methods:**

This retrospective study included patients diagnosed with PDAP at two centers between January 2015 and December 2025. Clinical data at admission were collected. Nutritional status was evaluated by the Prognostic Nutritional Index (PNI), the Controlling Nutritional Status (CONUT) score, and the Geriatric Nutritional Risk Index (GNRI). Receiver operating characteristic (ROC) curves and area under the curve (AUC) were used to compare the predictive ability of these three scores. Restricted cubic splines (RCS) were used to observe the dose–response relationship. Multivariable logistic regression was used to analyze the risk factors for treatment failure. Subgroup analyses were further performed to test the stability of the results. Net reclassification improvement (NRI) and integrated discrimination improvement (IDI) were used to evaluate the additional predictive value of the nutritional scores.

**Results:**

A total of 393 patients with PDAP were included. The rate of treatment failure was 30.79%. Among the three nutritional scores, PNI had the highest predictive value, with an AUC of 0.712. The AUCs of CONUT and GNRI were 0.681 and 0.633. All three nutritional scores showed linear dose–response relationships with treatment failure (*p* for nonlinearity: PNI = 0.242, CONUT = 0.748, GNRI = 0.952) and were identified as independent predictors in multivariable-adjusted analyses (all *p* < 0.001). Incremental analysis showed that adding PNI significantly improved the predictive performance over the baseline model (AUC: 0.627 to 0.723, *p* < 0.001), while CONUT showed moderate improvement (AUC: 0.627 to 0.700, *p* = 0.003) and GNRI did not (AUC: 0.627 to 0.662, *p* = 0.091). NRI and IDI confirmed the superior incremental value of PNI. Subgroup analyses showed no significant interactions (all *p* > 0.05).

**Conclusion:**

PNI demonstrated superior predictive and incremental value for treatment failure in PDAP compared with CONUT and GNRI, suggesting that it may serve as a practical tool for early risk stratification in this population.

## 1 Introduction

Peritoneal dialysis (PD) is a principal renal replacement therapy for patients with end-stage kidney disease (ESKD). Global utilization of PD varies markedly between countries and regions, being higher in developed nations and showing a stronger growth trend in developing ones (1–3). Peritoneal dialysis-associated peritonitis (PDAP) is a common complication among PD patients, with an incidence of approximately 0.1 to 0.4 episodes per patient-year across regions (4–7). A multicentre study found that 13.8% of patients required transfer to hemodialysis owing to PDAP treatment failure, and the mortality rate was 3.1% (8), substantially impairing quality of life and long-term survival.

Malnutrition is a frequent complication in patients with ESKD, closely linked to protein-energy wasting and microinflammation, and it substantially increases the risk of poor outcomes in patients on dialysis (9). Widely used composite tools include the Prognostic Nutritional Index (PNI), the Controlling Nutritional Status (CONUT) score and the Geriatric Nutritional Risk Index (GNRI); each is derived from routine laboratory and anthropometric measures and has seen broad application in the dialysis population. A cross-sectional study showed that PNI, CONUT, and GNRI were each independently associated with the malnutrition–inflammation score (MIS) in PD patients and could identify those at high risk of malnutrition (10). Subsequent studies have linked all three scores to all-cause mortality in dialysis patients (11–13). Of the three, a low PNI has been specifically associated with an increased risk of long-term infection in PD patients (14).

Most studies have focused on long-term outcomes such as all-cause mortality, with relatively few addressing the prediction of PDAP treatment failure. A small number of studies have examined associations between nutritional indicators and peritonitis, but most have been confined to single scores or to predicting peritonitis occurrence rather than treatment outcomes. A cohort study found that CONUT and PNI surpassed GNRI in predicting all-cause mortality and cardiovascular events among PD patients (15). Another investigation reported that PNI and CONUT also predicted PD technique failure effectively, while GNRI showed no significant association (16). Although GNRI showed the strongest correlation with MIS among the three scores (10), each nutritional index appeared to have its own strengths for different clinical outcomes. The comparative predictive value of these scores for PDAP treatment failure remains to be established. Therefore, this two-center study systematically compared the predictive performance of PNI, CONUT, and GNRI for PDAP treatment failure to identify the most appropriate nutritional tool for early clinical decision-making.

## 2 Materials and methods

### 2.1 Data source and patient selection

**Figure 1:**
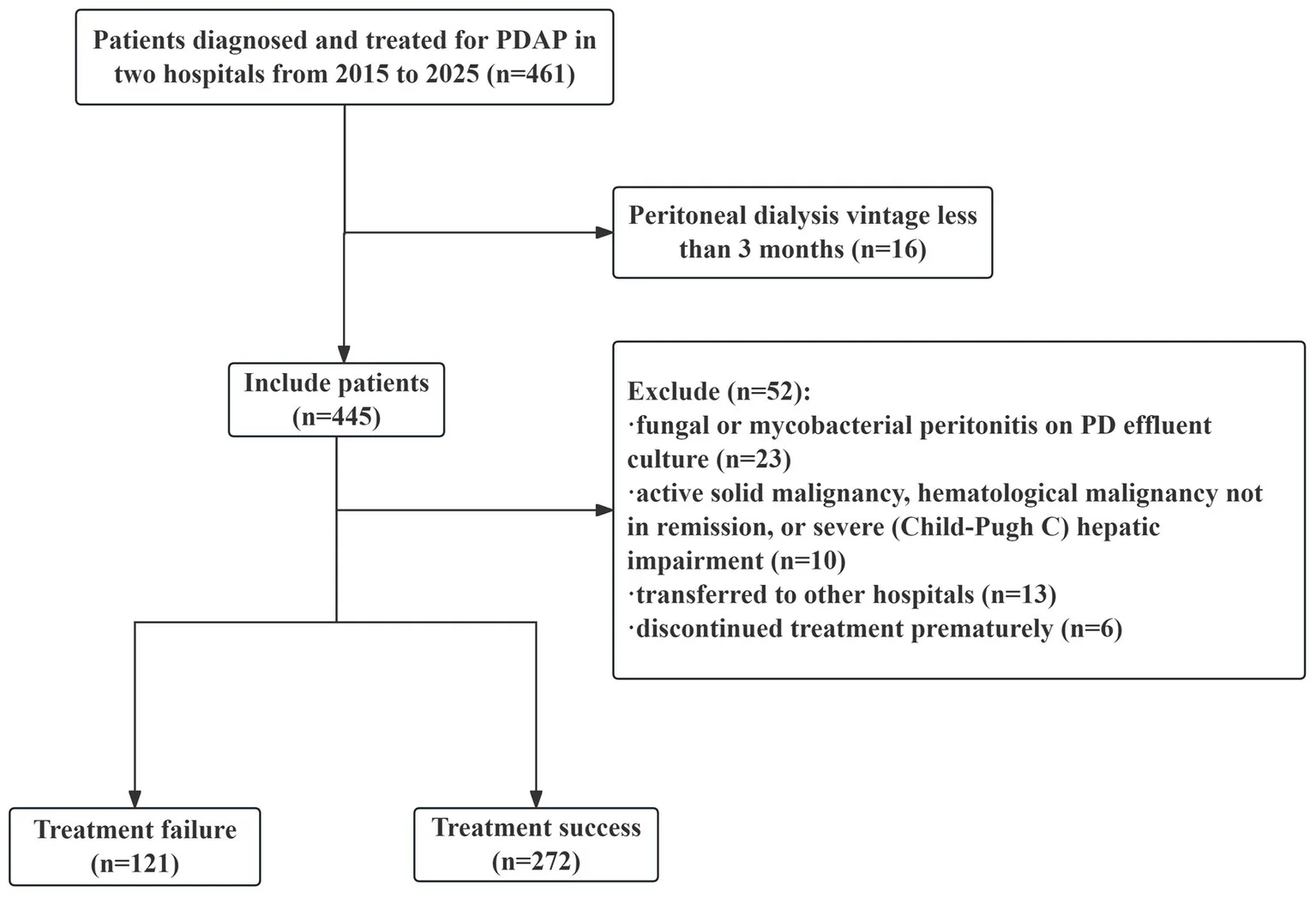
Flowchart of PDAP screening and selection process. PDAP, peritoneal dialysis-associated peritonitis.

This retrospective study enrolled patients with PDAP treated at Affiliated Hospital of Jiangsu University and Nanjing First Hospital between January 2015 and December 2025. Inclusion criteria were as follows: (1) patients with a diagnosis of PDAP according to International Society for Peritoneal Dialysis (ISPD) criteria (17); (2) peritoneal dialysis vintage ≥3 months. Exclusion criteria were as follows: 1. fungal or mycobacterial peritonitis on PD effluent culture; 2. active solid malignancy, hematological malignancy not in remission, or severe (Child-Pugh C) hepatic impairment (18); (3) missing data for any of the primary outcomes (peritonitis resolution, catheter removal, or death). The study protocol received approval from the research ethics committees of both centers (approval nos. KY2024K0502 and KY20220425-02), and written informed consent was waived because of the retrospective design. We included only the first PDAP episode for each patient and defined the date of PDAP diagnosis as the index date. The detailed process of patient selection is shown in Figure 1. The final cohort consisted of 393 patients, including 203 from the Affiliated Hospital of Jiangsu University and 190 from Nanjing First Hospital. Patient age ranged from 31 to 75 years. Nineteen patients were excluded because outcome data were unavailable; 13 were transferred to other hospitals, and six discontinued treatment prematurely.

### 2.2 Basic data and outcomes

Clinical data recorded on admission included demographic characteristics (sex, age), anthropometrics (height, weight), dialysis vintage, history of diabetes, peritoneal effluent culture (including detection of Gram-negative bacteria), time to first antibiotic administration, center and period. Blood tests performed within 24 h of admission comprised white blood cell count, neutrophil count, lymphocyte count, monocyte count, red blood cell count, hemoglobin, red blood cell distribution width, platelet count, C-reactive protein, total bilirubin, total cholesterol, triglycerides, uric acid, creatinine, blood urea nitrogen, serum potassium, serum chloride, serum phosphorus, serum sodium, glucose and albumin. Peritoneal effluent specimens were collected before antibiotic treatment and inoculated into aerobic and anaerobic blood culture bottles (BacT/ALERT, bioMérieux), followed by incubation at 35 °C. Positive cultures were subcultured onto blood agar and chocolate agar plates and incubated in 5% CO₂ at 35 °C for 24–48 h. Species identification was performed using matrix-assisted laser desorption/ionization time-of-flight mass spectrometry. Both centers followed the same microbiological protocol. We defined treatment failure according to the 2022 International Society for Peritoneal Dialysis peritonitis guideline (17) as catheter removal or all-cause mortality within 30 days after PDAP diagnosis. All patients were followed for at least 3 months from the index date. An independent nephrologist, blinded to the nutritional scores, adjudicated all outcome events.

### 2.3 Nutritional assessment

All variables required to calculate PNI, CONUT, and GNRI were obtained from routine blood tests performed on admission. Blood samples were collected before the first dose of antibiotics, so all three scores were available at the index date. To maintain laboratory consistency between the two centers during the 2015–2025 study period, both institutions participated in external quality assessment programs accredited by the National Center for Clinical Laboratories and performed periodic cross-validation assays. Nutritional status on admission was evaluated using three tools: PNI (19), CONUT score (20), and GNRI (21). The formulas for calculating three nutritional scores are as follows: PNI = serum albumin (g/L) + 5 × lymphocyte count (×10^9^/L). The CONUT score is derived from serum albumin, total cholesterol, and lymphocyte count; its grading criteria are provided in Table 1, and total cholesterol (mmol/L) × 38.67 = total cholesterol (mg/dL). GNRI = 1.489 × serum albumin (g/L) + 41.7 × (actual bodyweight/ideal bodyweight). Ideal bodyweight (kg) = 22 × height (m)^2^. If actual body weight exceeded ideal body weight, the weight ratio was set to 1.0. Ideal body weight was estimated using the formula 22 × height (m)^2^. This simplified formula is based on evidence that a body mass index of approximately 22 kg/m^2^ is associated with the lowest morbidity in Asian populations and has been widely used in nutritional studies involving patients receiving dialysis.

**Table 1:** Components and calculation of the Controlling Nutritional Status (CONUT) score.

Parameter	Calculation method			
Albumin (g/dL)	≥3.5	3.0–3.4	2.5–2.9	<2.5
score	0	2	4	6
Lymphocytes count (´10^9^/L)	≥1.600	1.200–1.599	0.800–1.199	<0.800
score	0	1	2	3
Total cholesterol (mg/dL)	≥180	140–179	100–139	<100
Score	0	1	2	3

### 2.4 Statistical analysis

All statistical analyses were performed using SPSS 26.0 and R 4.3.2. Three variables contained missing values: D-dimer (32.82%), procalcitonin (24.43%), and serum phosphorus (10.43%). We excluded D-dimer and procalcitonin from further analysis because more than 20% of their values were missing. Because the proportion of missing phosphorus values was relatively low, we performed multiple imputation using the random forest method in the mice package in R. Twenty imputed datasets were generated. The imputation model included all candidate predictors and the outcome variable under the assumption that the data were missing at random. We set the random seed to 12,345 to ensure reproducibility and used 5,000 burn-in iterations. Convergence was assessed by visual inspection of the trace plots. Serum phosphorus is not included in the calculation of PNI, CONUT, or GNRI and was not significantly associated with treatment failure in any analysis (all *p* > 0.05). Its imputation was therefore unlikely to have materially affected the comparison among the three nutritional indices. The Shapiro–Wilk test was used to examine whether continuous variables followed a normal distribution. Continuous variables with normal distribution were expressed as mean ± standard deviation and compared by an independent-samples *t*-test. Variables with skewed distribution were expressed as median and interquartile range, and the Mann–Whitney U test was used for comparison. Categorical variables were expressed as numbers and percentages, and the chi-square test was used. All tests were two-sided. A *p* value < 0.05 was considered statistically significant. ROC curves were constructed to evaluate the ability of PNI, CONUT, and GNRI to predict treatment failure in PDAP. The AUC of each score was calculated, and the AUCs were compared using DeLong’s test.

To determine whether the predictive performance of PNI was mainly related to its individual components, we compared PNI with serum albumin alone, lymphocyte count alone, and a logistic regression model in which albumin and lymphocyte count were entered as separate variables. Differences in the areas under the receiver operating characteristic curves were assessed using DeLong’s test. Likelihood ratio tests were used to assess the incremental predictive value of adding PNI to the baseline model and to compare the model containing PNI with the model containing albumin and lymphocyte count as separate variables. Restricted cubic spline models were used to examine the associations between each nutritional score and treatment failure. Knots were placed at the 10th, 50th, and 90th percentiles, and the median value of each score was used as the reference. The distribution of each predictor was overlaid on the spline curve to aid interpretation, and *p* values were reported for both the overall association and nonlinearity. RCS were adjusted for age, sex, dialysis vintage, and diabetes. The likelihood ratio test was used to test for nonlinearity. Univariate and multivariable logistic regression analyses were used to examine the associations between PNI, CONUT, GNRI, and treatment failure. Odds ratios were reported per 1-point increase in PNI, CONUT, and GNRI. All multivariable models were adjusted for study center to account for possible differences between the two institutions. The final model included 11 variables and 121 treatment-failure events, corresponding to approximately 11 events per variable.

To evaluate the incremental value of the models, First, a baseline model (Models 1) was established. Age, dialysis vintage, diabetes, and C-reactive protein were included as fixed covariates. Albumin was not included in this model because it is a component of all three nutritional scores. Then PNI, CONUT, and GNRI were added to the baseline model separately, forming three new models (Models 2, 3, and 4). ROC analysis, decision curve analysis, NRI, and IDI were used to compare these models. ROC analysis was used to evaluate discrimination. Decision curve analysis was used to assess clinical net benefit. NRI and IDI were used to determine whether the nutritional scores improved risk classification compared with the baseline model. Subgroup analyses were performed using the nutritional score with the best predictive performance. The score was treated as a continuous variable. The subgroups were stratified by sex, age, dialysis vintage, peritoneal effluent culture, and history of diabetes. For sensitivity analysis, the score was divided into high and low groups according to the median value, and the analyses were repeated. All subgroup models were adjusted for the same covariates, including C-reactive protein, glucose, total bilirubin, and triglycerides. Because the study covered 11 years, calendar period was included as a stratification variable in subgroup analyses and categorized as 2015–2020 or 2021–2025. All subgroup analyses were considered exploratory. The data supporting this study are available from the corresponding author upon reasonable request.

## 3 Results

### 3.1 Baseline characteristics

Of the 393 patients enrolled, 121 (30.79%) experienced treatment failure, including 116 cases (29.52%) of catheter removal and 5 cases (1.27%) of death. No overlap was observed between catheter removal and death. Compared with the success group, the failure group had significantly lower PNI and GNRI scores but a higher CONUT score (all *p* < 0.05). The two groups also differed significantly in dialysis vintage, peritoneal effluent culture, total bilirubin, triglycerides, glucose, lymphocyte count, and albumin (all *p* < 0.05). Table 2 summarizes the baseline characteristics.

**Table 2:** Comparison of general clinical data between treatment failure and treatment success groups.

Characteristic	Total (*n* = 393)	Treatment success (*n* = 272)	Treatment failure (*n* = 121)	*p*-value
General characteristics				
Sex (%)				0.293
Male	189 (48.09)	126 (46.32)	63 (52.07)	
Female	204 (51.91)	146 (53.68)	58 (47.93)	
Age (years)	55.00 (47.00, 65.00)	55.00 (45.00, 65.00)	57.00 (51.00, 65.00)	0.086
BMI (kg/m^2^)	22.17 (20.41, 23.54)	22.15 (20.28, 23.62)	22.20 (20.86, 23.31)	0.985
Dialysis vintage (months)	38.00 (20.00, 55.00)	36.00 (17.00, 50.00)	46.00 (26.00, 60.00)	<0.001
Time to antibiotic use (days)	1.00 (1.00, 3.00)	1.00 (1.00, 3.00)	1.00 (1.00, 3.00)	0.181
Diabetes (%)				0.353
No	282 (71.76)	199 (73.16)	83 (68.60)	
Yes	111 (28.24)	73 (26.84)	38 (31.40)	
Effluent (%)				<0.001
Negative	122 (31.04)	100 (36.76)	22 (18.18)	
Positive	271 (68.96)	172 (63.24)	99 (81.82)	
Gram-negative bacterial infection (%)				0.144
No	325 (82.70)	230 (84.56)	95 (78.51)	
Yes	68 (17.30)	42 (15.44)	26 (21.49)	
Center (%)				0.155
A	203 (51.65)	134 (49.26)	69 (57.02)	
B	190 (48.35)	138 (50.74)	52 (42.98)	
Period (%)				0.873
2015–2020	225 (57.25)	155 (56.99)	70 (57.85)	
2021–2025	168 (42.75)	117 (43.01)	51 (42.15)	
Laboratory measurements				
Monocyte count (10^9^/L)	0.50 (0.40, 0.60)	0.50 (0.40, 0.60)	0.50 (0.40, 0.70)	0.766
Red blood cell count (10^12^/L)	3.04 (2.54, 3.38)	2.98 (2.54, 3.39)	3.13 (2.61, 3.36)	0.348
RDW (%)	13.70 (12.80, 14.80)	13.60 (12.80, 14.83)	14.10 (12.90, 14.70)	0.356
Platelet count (10^9^/L)	194.00 (138.00, 265.00)	192.00 (138.00, 250.50)	205.00 (139.00, 286.00)	0.135
Hemoglobin (g/L)	90.00 (77.00, 102.00)	89.00 (76.00, 101.00)	91.00 (78.00, 103.00)	0.369
Total bilirubin (μmol/L)	4.30 (3.50, 5.60)	4.20 (3.38, 5.23)	4.50 (3.60, 6.30)	0.003
Blood urea nitrogen (mmol/L)	18.29 (14.07, 22.68)	18.58 (14.44, 22.92)	17.31 (13.65, 22.47)	0.229
Creatinine (μmol/L)	909.10 (768.00, 1,134.60)	908.00 (772.18, 1,139.53)	913.10 (754.60, 1,115.10)	0.448
Uric acid (μmol/L)	341.00 (314.00, 399.00)	340.50 (316.00, 404.25)	341.00 (307.00, 393.00)	0.379
Glucose (mmol/L)	5.28 (4.49, 6.27)	5.11 (4.44, 5.92)	5.82 (4.73, 7.23)	<0.001
Triglyceride (mmol/L)	1.49 (1.19, 1.86)	1.38 (1.16, 1.77)	1.66 (1.29, 1.95)	<0.001
Total cholesterol (mmol/L)	4.06 (3.66, 4.79)	4.04 (3.64, 4.76)	4.08 (3.73, 4.99)	0.259
Potassium (mmol/L)	3.68 (3.44, 4.02)	3.71 (3.47, 4.06)	3.64 (3.43, 3.93)	0.157
Sodium (mmol/L)	136.30 (134.40, 139.20)	136.25 (134.57, 139.10)	136.50 (134.20, 139.20)	0.558
Chloride (mmol/L)	98.60 (95.80, 102.10)	98.65 (95.77, 102.50)	98.60 (96.10, 101.70)	0.206
Phosphorus (mmol/L)	1.49 (1.24, 1.81)	1.49 (1.24, 1.79)	1.49 (1.23, 1.81)	0.881
Inflammatory parameters				
White blood cell count (10^9^/L)	8.30 (6.30, 10.90)	8.30 (6.30, 10.83)	8.30 (6.20, 10.90)	0.987
Neutrophil count (10^9^/L)	6.30 (4.60, 9.40)	6.35 (4.50, 9.50)	6.10 (4.70, 9.30)	0.926
Lymphocyte count (10^9^/L)	0.90 (0.70, 1.30)	1.00 (0.70, 1.50)	0.80 (0.60, 1.10)	<0.001
C-reactive protein (mg/L)	49.30 (26.70, 94.10)	47.15 (26.85, 87.23)	59.80 (26.50, 108.10)	0.165
Nutritional parameters				
ALB (g/L)	34.30 (31.30, 37.40)	35.10 (32.30, 37.95)	32.30 (28.30, 35.40)	<0.001
PNI	39.30 (35.90, 42.60)	39.90 (37.58, 43.52)	36.50 (32.50, 40.30)	<0.001
CONUT	4.00 (3.00, 6.00)	4.00 (2.00, 5.00)	5.00 (4.00, 7.00)	<0.001
GNRI	90.87 (86.28, 95.75)	91.58 (88.23, 96.55)	89.50 (82.33, 93.79)	<0.001

Data are presented as *N* (%) or Median (IQR). BMI, body mass index; A, Affiliated Hospital of Jiangsu University; B, Nanjing First Hospital; RDW, red blood cell distribution width; ALB, albumin; PNI, Prognostic Nutritional Index; CONUT, Controlling Nutritional Status; GNRI, Geriatric Nutritional Risk Index. Continuous variables were compared by *t*-test (normal, mean ± SD) or Mann–Whitney *U* test (skewed, median[IQR]); categorical variables by *χ*^2^ test (*n*[%]). Denominators: effective n per group (*t*-test), rank-sum n per group (*U* test), or total *n* per group (*χ*^2^). Two-sided, *p* < 0.05 significant. See Statistical analysis for details.

### 3.2 Association between nutritional status and treatment failure in PDAP

**Figure 2:**
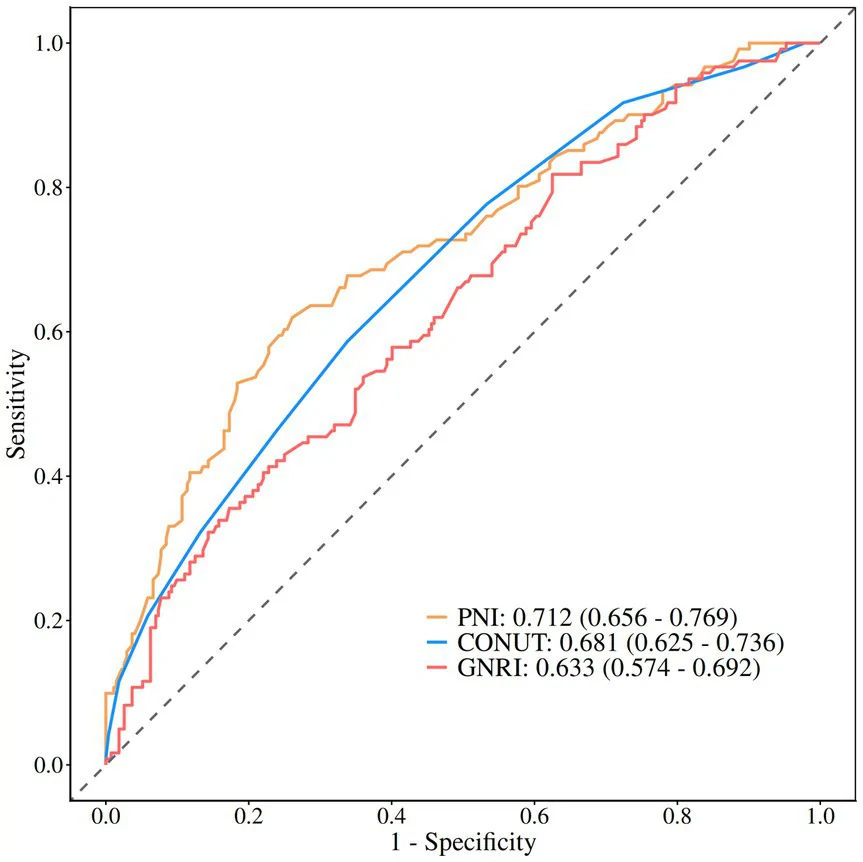
Receiver operating characteristic (ROC) curves of PNI, CONUT, and GNRI for predicting treatment failure. PNI, Prognostic Nutritional Index; CONUT, Controlling Nutritional Status score; GNRI, Geriatric Nutritional Risk Index.

ROC curves were constructed to assess the predictive performance of the three nutritional indices. The AUCs for PNI, CONUT, and GNRI were 0.712 (95% CI: 0.656–0.769), 0.681 (95% CI: 0.625–0.736), and 0.633 (95% CI: 0.574–0.692), respectively (Figure 2). DeLong’s test indicated that the AUC for PNI was significantly larger than that for CONUT (*Z* = 2.128, *p* = 0.033) and GNRI (*Z* = 5.508, *p* < 0.001).

**Figure 3:**
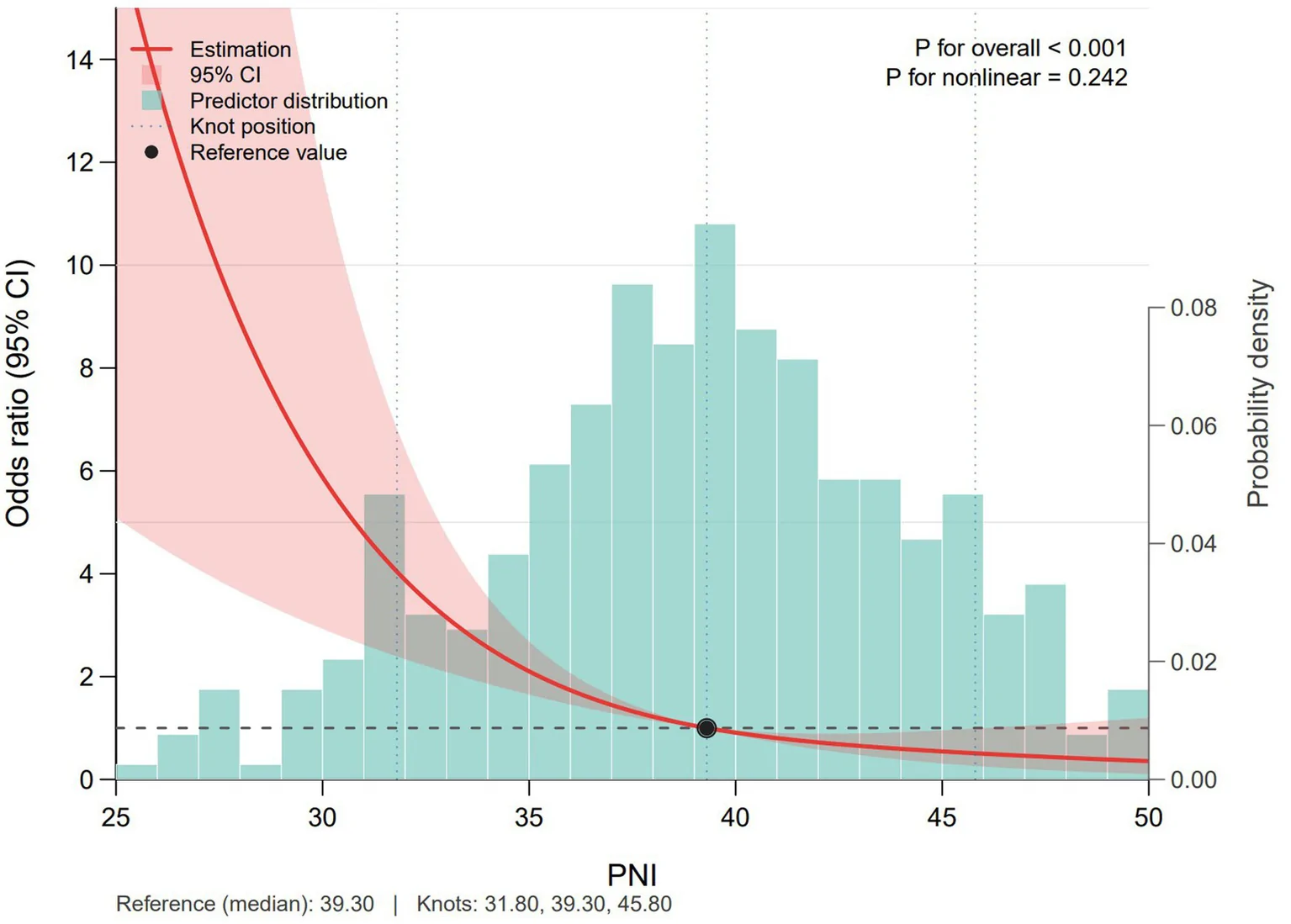
Analysis of the relationship between PNI and treatment failure: restricted cubic spline plot and frequency distribution histogram. PNI, Prognostic Nutritional Index.

**Figure 4:**
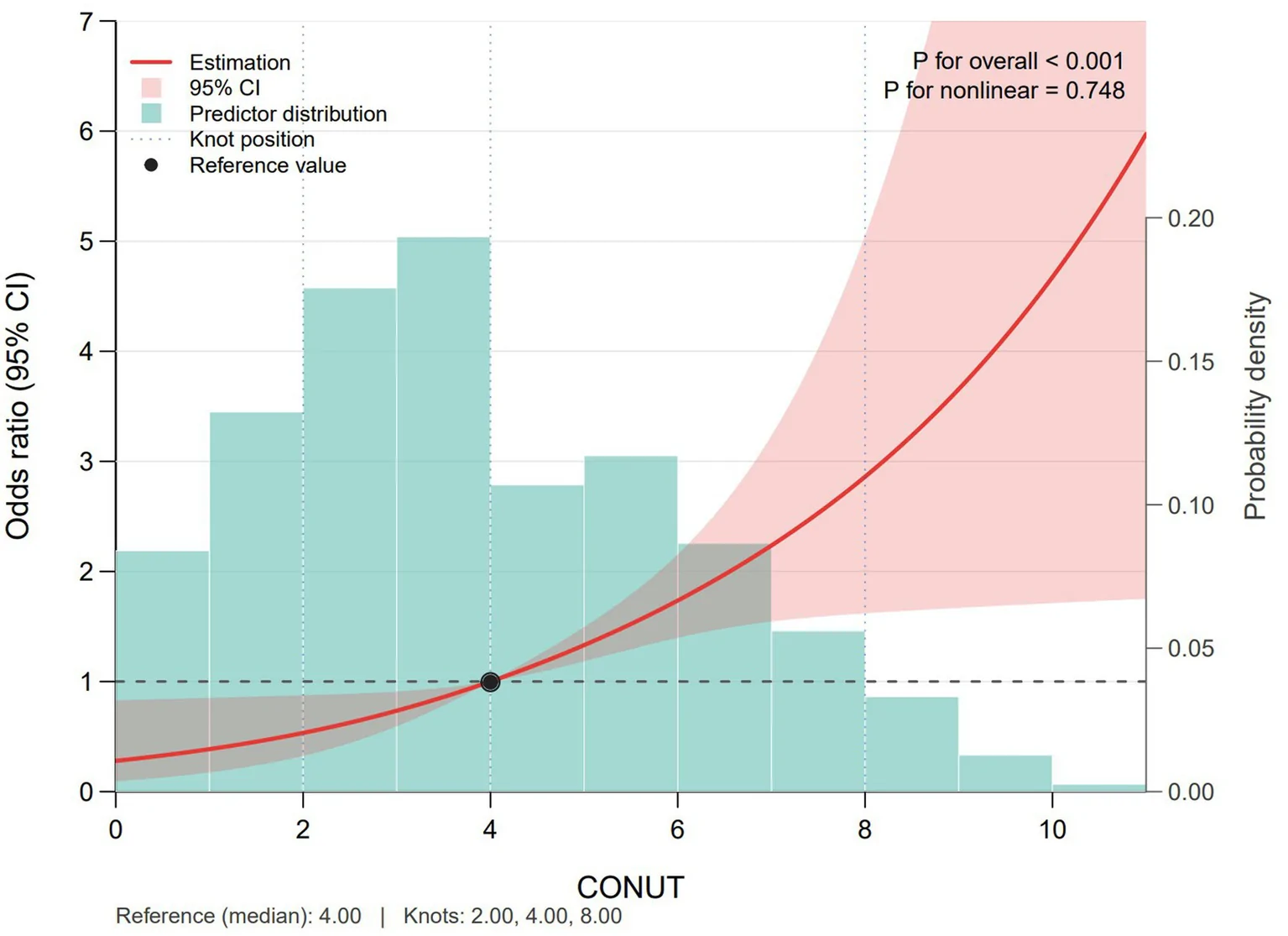
Analysis of the relationship between CONUT and treatment failure: restricted cubic spline plot and frequency distribution histogram. CONUT, Controlling Nutritional Status score.

**Figure 5:**
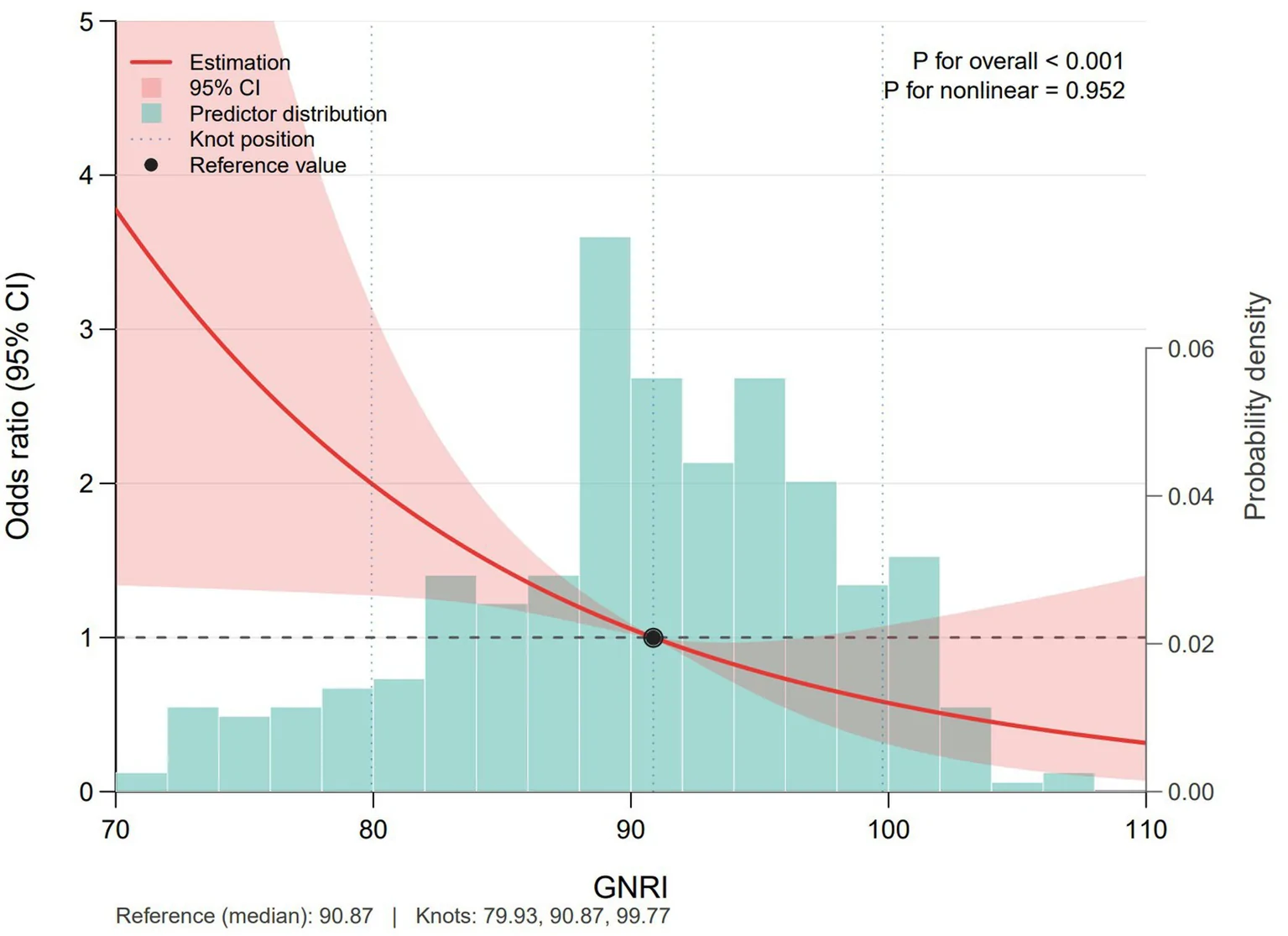
Analysis of the relationship between GNRI and treatment failure: restricted cubic spline plot and frequency distribution histogram. GNRI, Geriatric Nutritional Risk Index.

We compared the predictive performance of PNI with that of serum albumin, lymphocyte count, and the combination of albumin and lymphocyte count entered as separate variables in a logistic regression model. The AUCs for albumin, lymphocyte count, and their combined use were 0.660, 0.661, and 0.734, respectively. DeLong’s test showed that PNI had significantly better discriminatory performance than albumin alone (*Z* = 4.493, *p* < 0.001). No significant difference was found between PNI and lymphocyte count alone (*Z* = 1.602, *p* = 0.109) or between PNI and the combined use of albumin and lymphocyte count (*Z* = 1.869, *p* = 0.062). We performed a likelihood ratio test to compare the baseline model plus PNI with the baseline model plus albumin and lymphocyte count as separate variables. The model containing albumin and lymphocyte showed better overall fit than the model containing PNI (*χ*^2^ = 7.844, *p* = 0.005). We used RCS analysis to examine the dose–response relationship between each nutritional index and treatment failure. No significant nonlinearity was detected for any of the three scores, with P for nonlinearity of 0.242, 0.748, and 0.952 (Figures 3–5). Univariate analysis identified lower PNI, higher CONUT, and lower GNRI as significant predictors of treatment failure (all *p* < 0.05). Other baseline variables that showed between-group differences (Table 2) were also associated with the outcome in univariate analysis (Table 3). After adjustment for confounders in multivariate models (Table 4), all three scores remained independent predictors: PNI (OR = 0.854, 95% CI: 0.806–0.905, *p* < 0.001), CONUT (OR = 1.298, 95% CI: 1.152–1.461, *p* < 0.001), and GNRI (OR = 0.943, 95% CI: 0.911–0.976, *p* < 0.001). At the optimal PNI cutoff determined by the Youden index (PNI ≤ 37.7), the sensitivity and specificity for predicting treatment failure were 61.98 and 73.90%.

**Table 3:** Univariate logistic regression analysis of treatment failure.

Characteristic	OR (95% CI)	*p-*value
General characteristics		
Sex		
Male	Reference	
Female	0.79 (0.52–1.22)	0.293
Age (years)	1.02 (1.00–1.04)	0.063
BMI (kg/m^2^)	1.00 (0.91–1.10)	0.938
Dialysis vintage (months)	1.02 (1.01–1.02)	<0.001
Time to antibiotic use (days)	1.07 (0.98–1.17)	0.133
Diabetes		
No	Reference	
Yes	1.25 (0.78–1.99)	0.354
Effluent		
Negative	Reference	
Positive	2.62 (1.55–4.42)	<0.001
Gram-negative bacterial infection		
No	Reference	
Yes	1.50 (0.87–2.58)	0.145
Center (%)		
A	Reference	
B	0.73 (0.47–1.13)	0.156
Period		
2015–2020	Reference	
2021–2025	0.96 (0.63–1.49)	0.873
Laboratory measurements		
Monocyte count (10^9^/L)	0.78 (0.25–2.40)	0.665
Red blood cell count (10^12^/L)	1.22 (0.89–1.67)	0.221
RDW (%)	1.03 (0.91–1.16)	0.676
Platelet count (10^9^/L)	1.00 (1.00–1.00)	0.147
Hemoglobin (g/L)	1.00 (0.99–1.02)	0.434
Total bilirubin (μmol/L)	1.12 (1.03–1.22)	0.008
Blood urea nitrogen (mmol/L)	0.99 (0.95–1.02)	0.366
Creatinine (μmol/L)	1.00 (1.00–1.00)	0.216
Uric acid (μmol/L)	1.00 (1.00–1.00)	0.691
Glucose (mmol/L)	1.41 (1.23–1.62)	<0.001
Triglyceride (mmol/L)	2.03 (1.34–3.08)	<0.001
Total cholesterol (mmol/L)	1.08 (0.87–1.34)	0.462
Potassium (mmol/L)	0.85 (0.59–1.23)	0.390
Sodium (mmol/L)	0.98 (0.92–1.04)	0.503
Chloride (mmol/L)	0.96 (0.91–1.01)	0.099
Phosphorus (mmol/L)	1.13 (0.69–1.84)	0.620
Inflammatory parameters		
White blood cell count (10^9^/L)	0.99 (0.94–1.05)	0.835
Neutrophil count (10^9^/L)	0.98 (0.93–1.03)	0.470
Lymphocyte count (10^9^/L)	0.18 (0.10–0.35)	<0.001
C-reactive protein (mg/L)	1.00 (1.00–1.01)	0.123
Nutritional parameters		
ALB (g/L)	0.87 (0.83–0.92)	<0.001
PNI	0.85 (0.81–0.89)	<0.001
CONUT	1.35 (1.22–1.50)	<0.001
GNRI	0.93 (0.91–0.96)	<0.001

BMI, body mass index; A, Affiliated Hospital of Jiangsu University; B, Nanjing First Hospital; RDW, red blood cell distribution width; ALB, albumin; PNI, Prognostic.

**Table 4:** Multivariable stepwise regression.

Model	PNI	CONUT	GNRI
Model A			
OR (95% CI)	0.846 (0.805–0.889)	1.354 (1.221–1.501)	0.934 (0.907–0.963)
*p*-value	<0.001	<0.001	<0.001
Model B			
OR (95% CI)	0.853 (0.810–0.898)	1.321 (1.187–1.470)	0.939 (0.911–0.969)
*p*-value	<0.001	<0.001	<0.001
Model C			
OR (95% CI)	0.854 (0.806–0.905)	1.298 (1.152–1.461)	0.943 (0.911–0.976)
*p*-value	<0.001	<0.001	<0.001

PNI, Prognostic Nutritional Index; CONUT, Controlling Nutritional Status score; GNRI, Geriatric Nutritional Risk Index; Data are presented as odds ratio, 95% CI (confidence intervals), and P value. Model A adjusted for none. Model B adjusted for sex, age, dialysis vintage, diabetes. Model C adjusted for sex, age, dialysis vintage, diabetes, effluent culture, C-reactive protein, blood glucose, total bilirubin, triglycerides, center.

### 3.3 Incremental predictive value of adding nutritional scores

**Figure 6:**
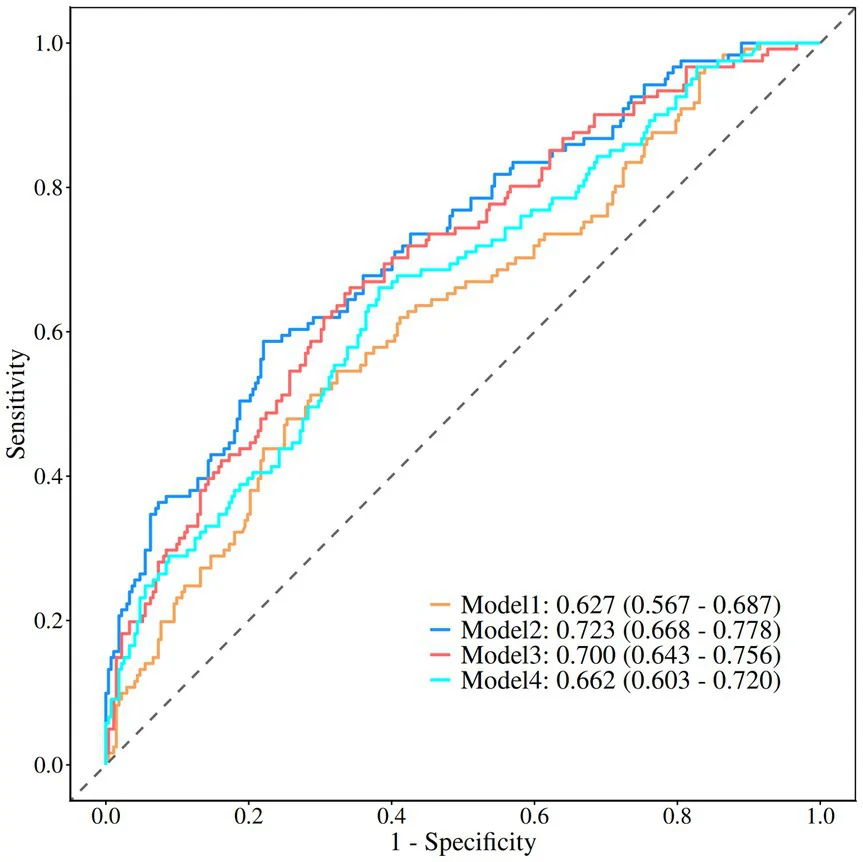
Receiver operating characteristic (ROC) curves of Model 1, Model 2, Model 3, Model 4 for predicting treatment failure. Model 1 was the baseline model, with age, dialysis vintage, diabetes, and C-reactive protein included as fixed covariates. Subsequently, PNI, CONUT, and GNRI were added to the baseline model separately to generate Models 2, 3, and 4. PNI, Prognostic Nutritional Index; CONUT, Controlling Nutritional Status score; GNRI, Geriatric Nutritional Risk Index.

**Figure 7:**
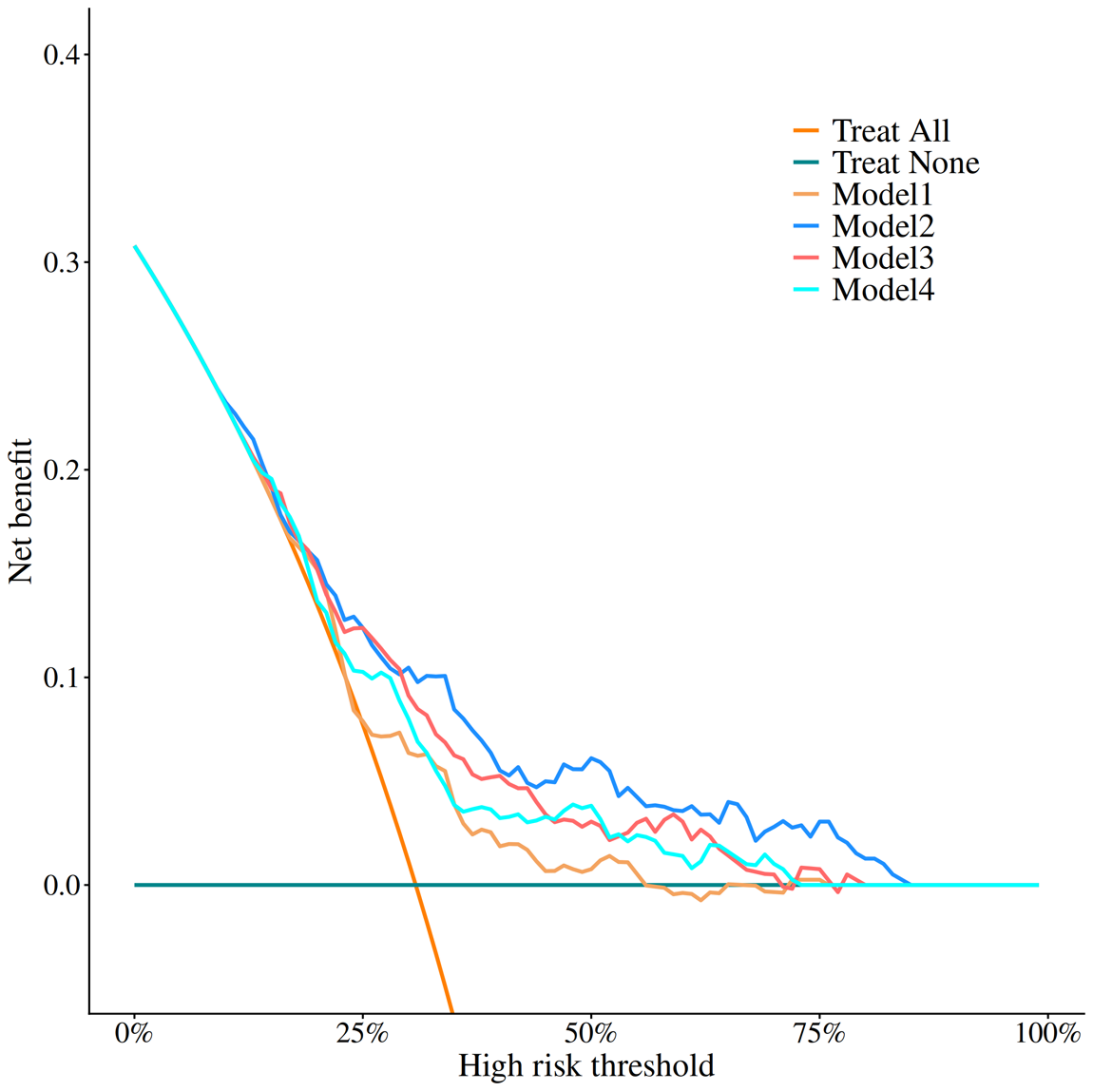
Decision curve analysis (DCA) of Model 1, Model 2, Model 3, Model 4 for predicting treatment failure. Model 1 was the baseline model, with age, dialysis vintage, diabetes, and C-reactive protein included as fixed covariates. Subsequently, PNI, CONUT, and GNRI were added to the baseline model separately to generate Models 2, 3, and 4. PNI, Prognostic Nutritional Index; CONUT, Controlling Nutritional Status score; GNRI, Geriatric Nutritional Risk Index.

Model 1 (baseline), which included age, dialysis vintage, diabetes, and C-reactive protein, achieved an AUC of 0.627. Adding PNI, CONUT, or GNRI to Model 1 produced Models 2, 3, and 4, with AUCs of 0.723, 0.700, and 0.662. Compared with Model 1, Model 2 (*Z* = 3.464, *p* < 0.001) and Model 3 (*Z* = 2.923, *p* = 0.003) showed significantly better discrimination, whereas Model 4 did not (*Z* = 1.688, *p* = 0.091; Figure 6). Decision curve analysis further indicated that Model 2 offered the highest net benefit across the 25–75% threshold probability range (Figure 7). However, all three extended models significantly improved continuous net reclassification improvement and integrated discrimination improvement compared with the baseline model (all *p* < 0.05; Table 5). Model 2 showed the greatest incremental improvement, with a continuous NRI of 0.54 and an IDI of 0.11, indicating that adding PNI to the baseline variables improved both risk reclassification and discrimination.

**Table 5:** Comparison of incremental predictive value of the models.

Model	Continuous NRI (95% CI)	*p*-value	IDI (95% CI)	*p*-value
Model 1	Reference		Reference	
Model 2	0.54 (0.33–0.75)	<0.001	0.11 (0.07–0.14)	<0.001
Model 3	0.51 (0.30–0.71)	<0.001	0.07 (0.04–0.09)	<0.001
Model 4	0.27 (0.06–0.48)	0.011	0.04 (0.02–0.06)	<0.001

Model 1 included age, dialysis vintage, diabetes, and C-reactive protein; Models 2–4 additionally included PNI, CONUT score, and GNRI. PNI, Prognostic Nutritional Index; CONUT, Controlling Nutritional Status score; GNRI, Geriatric Nutritional Risk Index; NRI, Net Reclassification Improvement; IDI, Integrated Discrimination Improvement.

### 3.4 Subgroup analysis of the association between PNI and treatment failure

**Figure 8:**
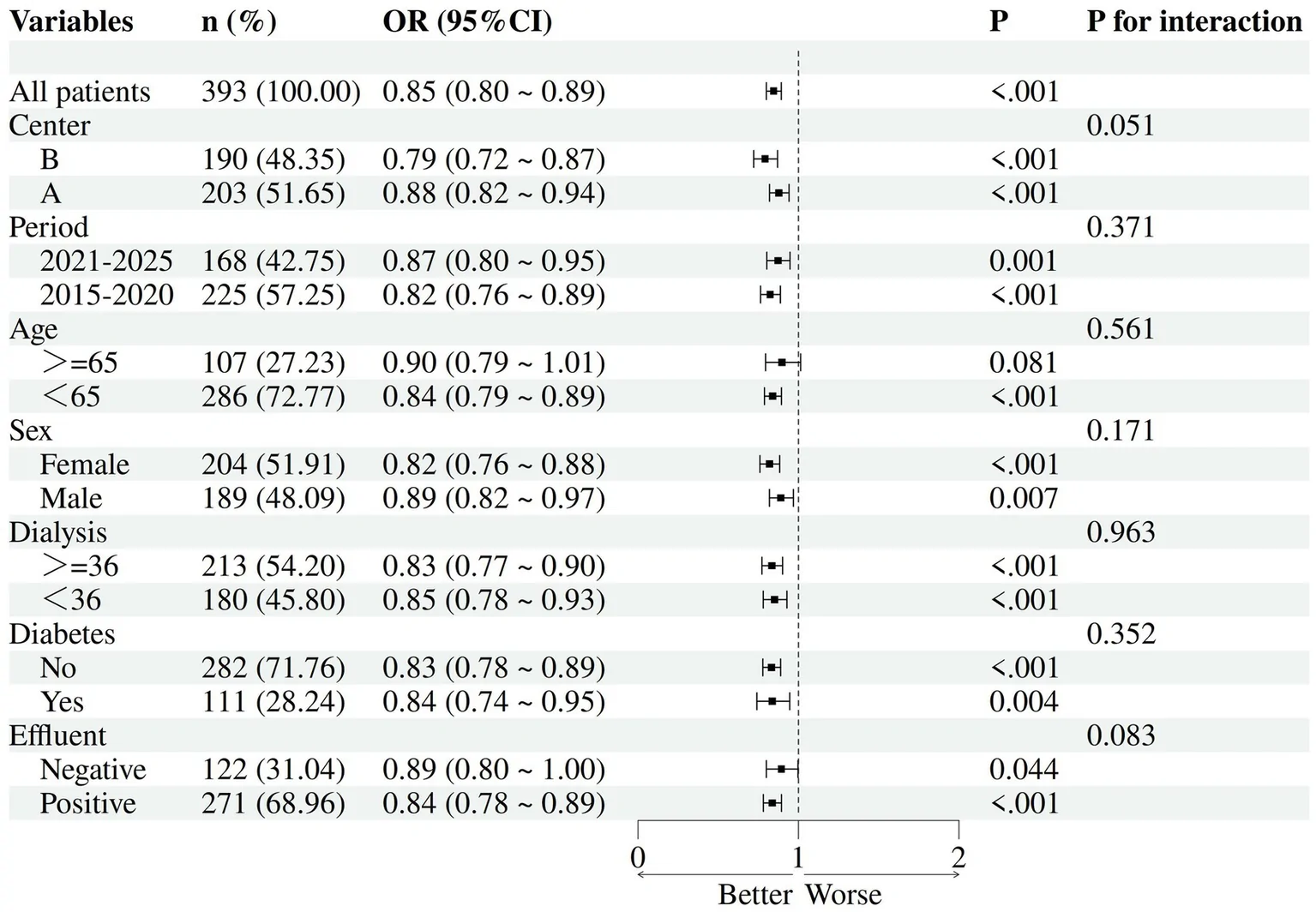
Subgroup analysis of associations between PNI (as a continuous variable) and treatment failure. **(A)** Affiliated Hospital of Jiangsu University; **(B)** Nanjing First Hospital; PNI, Prognostic Nutritional Index; OR, Odds Ratio; 95% CI, 95% Confidence Interval.

**Figure 9:**
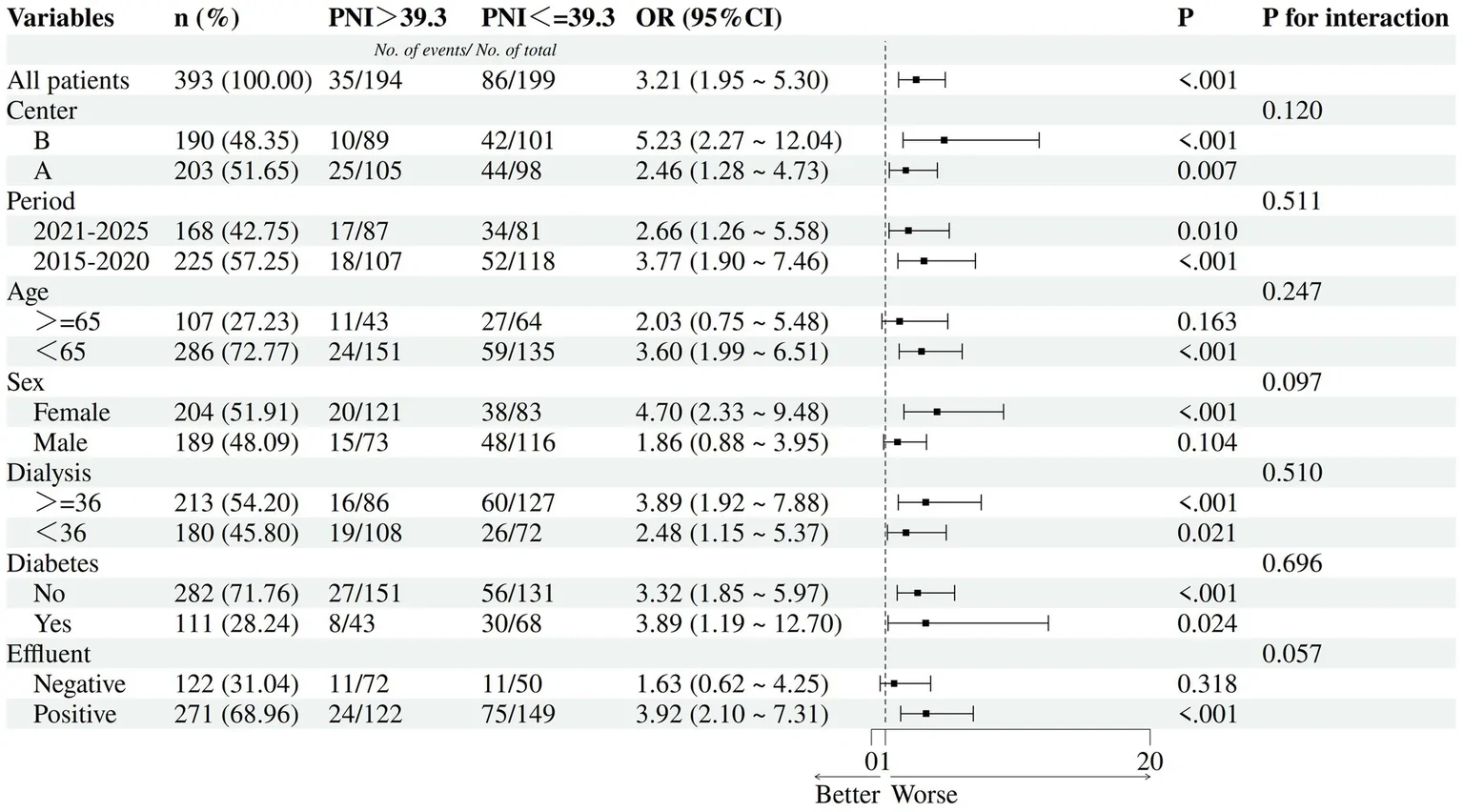
Subgroup analysis of associations between median PNI (dichotomized at the median) and treatment failure. **(A)** Affiliated Hospital of Jiangsu University; **(B)** Nanjing First Hospital; PNI, prognostic nutritional index; OR, odds ratio; 95% CI, 95% confidence interval.

We stratified patients based on center, period, sex, age, dialysis vintage, peritoneal effluent culture, diabetes history. No significant interaction was found between PNI and treatment failure in any subgroup, irrespective of whether PNI was analyzed as a continuous variable (Figure 8) or as a binary variable split at the median (Figure 9). Although the point estimates varied across some subgroups, none of the interaction tests reached statistical significance. All interaction *p* values were greater than 0.05, and there was no evidence that the association between PNI and treatment failure differed according to study center, calendar period, sex, age, dialysis vintage, effluent culture result, or history of diabetes.

## 4 Discussion

In this study, we compared PNI, CONUT, and GNRI in patients with PDAP. Among these three nutritional scores, PNI showed the strongest association with treatment failure. Patients in the treatment failure group had lower PNI and GNRI, but higher CONUT scores. After adjustment for clinical factors, PNI was still independently associated with treatment failure. When PNI was added to the baseline model, the predictive ability of the model also improved. The subgroup analyses showed similar results in different patient groups. These nutritional scores, calculated from routine laboratory data at PDAP diagnosis, are intended to assist clinicians in early risk stratification for treatment failure, potentially guiding more intensive monitoring or timely intervention. The three nutritional indices should not be interpreted as pure measures of chronic nutritional status. Serum albumin, lymphocyte count, and total cholesterol were measured during acute episodes of PDAP and may change in response to systemic inflammation. These scores are therefore better viewed as markers of the combined nutritional and inflammatory state at diagnosis. Their predictive value may reflect the severity of infection and the acute inflammatory response, as well as the patient’s underlying nutritional reserve.

Previous studies have shown that malnutrition is associated with poor prognosis in patients undergoing peritoneal dialysis (22–24). Our results suggest that this association also exists in patients with PDAP. There are several possible explanations. First, malnutrition is often accompanied by impaired immune function. In this situation, the body may have a weaker ability to clear pathogens from the peritoneal cavity. Second, patients with poor nutritional status may have less reserve when they experience an infection. They may therefore be less able to tolerate the inflammatory response caused by PDAP. Third, low albumin and other reduced drug-binding proteins may affect antibiotic pharmacokinetics (25). This may influence the effect of anti-infective treatment. These factors may partly explain why nutritional scores were related to treatment failure in our cohort. The culture-negative rate was 31%, which was consistent with rates reported in previous studies of peritoneal dialysis-associated peritonitis. Possible explanations include a low microbial burden and the presence of fastidious organisms. Culture status was unlikely to directly affect PNI, CONUT, or GNRI because all three scores are based on serum variables, and the primary findings remained consistent after adjustment for culture results.

Longer dialysis vintage and culture-positive status were more common in the treatment-failure group. These characteristics may reflect cumulative uremic exposure and greater infection severity, respectively, and may contribute to poor nutritional status or coexist with it. Although we adjusted the analyses for these variables, the present study cannot determine whether impaired nutritional status is an independent predictor, a mediator of disease severity, or a marker of other underlying pathological processes.

A key point of this study is that we compared the three scores directly. PNI, CONUT, and GNRI were all independently associated with treatment failure. PNI and GNRI were negatively associated with treatment failure, while CONUT was positively associated with treatment failure. This means that worse nutritional status was related to a higher risk of poor treatment response, no matter which score was used. However, PNI showed the best predictive performance. PNI is calculated from serum albumin and lymphocyte count. These two indicators are simple and can be obtained from routine blood tests. Albumin is usually regarded as a nutritional marker, but it also reflects inflammation and general disease condition. Lymphocyte count is related to immune function. For PDAP, this may be especially important, because the outcome of infection depends not only on antibiotics but also on the patient’s immune response. Some studies have also reported that T-cell subsets may be involved in the progression of peritoneal dialysis-related complications (26). This also supports the value of immune-related indicators in this population. PNI and CONUT both include albumin and lymphocyte count, but PNI performed better than CONUT in our study. One possible reason is that CONUT also includes cholesterol. Cholesterol may reflect long-term nutritional status, but it is not always stable in patients on peritoneal dialysis. It may be affected by statin use, residual renal function, dietary intake, inflammation, and other metabolic factors. During an acute infection such as PDAP, the value of cholesterol as a nutritional marker may be further weakened. This may explain why CONUT did not perform as well as PNI. Similar findings have been reported in other diseases. For example, PNI was better than CONUT in predicting contrast-induced acute kidney injury (27). In prostate cancer, PNI, but not CONUT, was an independent predictor of progression-free survival (28). GNRI includes serum albumin and the ratio of actual body weight to ideal body weight. It has been used to predict mortality and cardiovascular events in patients undergoing peritoneal dialysis (29). However, in our cohort, GNRI was weaker than PNI in predicting treatment failure. This may be related to the body weight component of GNRI. In patients with PDAP, acute infection may cause changes in fluid status, appetite, and body composition. Body weight may also be affected by dialysis fluid and volume overload. Therefore, weight-based nutritional assessment may not be accurate enough during an acute episode. In addition, GNRI does not include an immune-related parameter. This may be a limitation when it is used to predict the outcome of infection. These three indices are also not entirely independent. Serum albumin is included in all three scores, while both PNI and CONUT include lymphocyte count. This overlap may partly explain the better performance of PNI, which is a direct composite of albumin and lymphocyte count, rather than indicating a distinct biological pathway. From a practical perspective, PNI remains a simple, readily available composite measure that may be easier to apply at the bedside than a model that requires the two components to be entered and interpreted separately.

After PNI was added to the baseline model, the model performance improved obviously. The AUC increased from 0.627 to 0.723. The model with PNI also had the highest net benefit in decision curve analysis when the threshold probability ranged from 25 to 75%. NRI and IDI also showed significant improvement after adding PNI. These results indicate that PNI provided extra predictive information beyond age, dialysis vintage, diabetes, and CRP. In other words, PNI may help identify high-risk patients more accurately at the time of PDAP diagnosis. The relationship between PNI and treatment failure was stable in subgroup analyses. All interaction *p* values were greater than 0.05, indicating no evidence of heterogeneity across subgroups by center, period, sex, age, dialysis vintage, effluent culture results, or diabetes history. This suggests that the predictive value of PNI was not strongly affected by these clinical characteristics. Since PNI only requires albumin and lymphocyte count, it is easy to use in daily clinical practice. The model incorporating PNI achieved an AUC of 0.723. Although this value was higher than those of the models incorporating CONUT or GNRI, the model showed only modest discrimination. At the optimal cutoff of the model-predicted probability, sensitivity was 58.68%, and specificity was 77.94%. Decision curve analysis showed a net clinical benefit over only a limited range of threshold probabilities. The model should therefore be used as a supplementary screening tool rather than as the sole basis for treatment decisions. A high-risk result may alert clinicians to the need for closer observation or earlier intervention, but decisions concerning catheter removal, treatment escalation, or other major clinical measures should still be based on a comprehensive clinical assessment.

This study has some limitations. First, the patients were from two peritoneal dialysis centers in China. Therefore, the results may not be directly applicable to other populations. Both participating centers are located in Jiangsu Province, which may limit the geographic generalizability of the findings, and external validation in larger and more diverse populations is needed. Second, this was a retrospective study, and some unmeasured confounding factors may still exist. Third, we only evaluated nutritional scores at admission. We did not analyze dynamic changes in nutritional status during treatment. In addition, the laboratory variables used to calculate the three indices may be influenced by the acute inflammatory response during PDAP, so these scores should be interpreted as nutrition-inflammation markers rather than isolated measures of nutritional status. Fourth, the optimal cutoff values of PNI, CONUT, and GNRI for PDAP have not been clearly established. More prospective and multicenter studies are needed to confirm our findings and determine clinically useful thresholds. Finally, this study does not perform formal calibration assessment; we suggest that future external validation studies include calibration metrics to evaluate these scores.

In conclusion, PNI, CONUT, and GNRI were all independently associated with treatment failure in patients with PDAP. Among the three nutritional indices examined, PNI showed the strongest association with treatment failure. However, given the modest difference between PNI and CONUT, these findings should be interpreted cautiously and do not support causal inference. Although the model containing albumin and lymphocyte count as separate variables showed better statistical fit, PNI achieved similar discriminatory performance and performed better than albumin alone. PNI may therefore serve as a simple supplementary tool for early risk stratification in patients with PDAP.

## Data Availability

The raw data supporting the conclusions of this article will be made available by the authors, without undue reservation.
